# The Distribution of *K. pneumoniae* in Different Specimen Sources and Its Antibiotic Resistance Trends in Sichuan, China From 2017 to 2020

**DOI:** 10.3389/fmed.2022.759214

**Published:** 2022-02-15

**Authors:** Jie Zhang, Dan Li, Xiangning Huang, Shanshan Long, Hua Yu

**Affiliations:** ^1^Department of Laboratory Medicine, Sichuan Provincial People's Hospital, University of Electronic Science and Technology of China, Chengdu, China; ^2^School of Medicine, University of Electronic Science and Technology of China, Chengdu, China; ^3^Department of Laboratory Medicine, Medical Center Hospital of Qionglai City, Chengdu, China

**Keywords:** antimicrobial resistance, *Klebsiella pneumoniae*, surveillance, carbapenem-resistant, antimicrobial susceptibility

## Abstract

**Objective:**

We aim to analyze the distribution of *Klebsiella pneumoniae* in different specimen sources and its antibiotic resistance trends from the Antimicrobial Resistant Investigation Network of Sichuan Province (ARINSP) between 2017 and 2020.

**Methods:**

According to the monitoring scheme, each participating hospital identified the bacteria and performed antimicrobial susceptibility tests using approved procedures. The data of non-repetitive isolates collected from outpatients and inpatients were submitted to ARINSP. The WHONET 5.6 software was used to analyze the results according to the Clinical and Laboratory Standards Institute (CLSI).

**Results:**

Between 2017 and 2020, 833,408 non-repetitive clinical isolates of bacteria were isolated in total. The bacterial strains isolated from sputum and broncho-alveolar lavage accounted for 48.7, 56.4, 49.2, and 43.7% from 2017 to 2020 respectively, among all sources. The number of *Klebsiella pneumoniae* isolates from sputum and broncho-alveolar lavage increased from 18,809 in 2018, 19,742 in 2019, to 19,376 in 2020, playing a predominant role among all specimens. Meropenem-resistant *K. pneumoniae* occurrences (5.7% in 2017, 7.3% in 2018, 8.0% in 2019, and 7.5% in 2020) remained highest among carbapenems, and increased slightly over time. The resistance rate to tigecycline remained lowest, and declined from 2.4% in 2017, to 0.4% in 2018, and from 0.7% in 2019, to 0.6% in 2020.

**Conclusion:**

The overall resistance rates of *Klebsiella pneumoniae* to carbapenems increased in Sichuan Province, giving a significant challenge to control *K. pneumoniae* related infections. Tigecycline has retained activity to against *K. pneumoniae*. Ongoing surveillance is essential. It can help for implementing intervention programs to reduce the occurrence of antimicrobial resistance and to provide with a rational use of antimicrobials.

## Introduction

*Klebsiella pneumoniae* (*K. pneumoniae*) is an increasingly important gram-negative pathogen that can cause serious infections. According to the 2019 Antimicrobial Resistant Threats Report (1) from the Centers for Disease Control and Prevention (CDC), carbapenem-resistant Enterobacteriaceae (CRE), which commonly cause hard to treat infections among patients, were listed as “urgent threats” to public health. In the European Union and China, carbapenem-resistant *Klebsiella pneumoniae* (CRKP) strains account for ~64–87.8% of clinical CRE infections (2–4). Infections caused by CRE (CRKP most frequently) are associated with higher mortality (1) and increased healthcare burden (5, 6).

Nevertheless, due to the difference in resistance mechanisms, the resistance patterns in bacteria are various in different regions (3, 7–9). Carbapenemase is the primary carbapenem resistance mechanism among *K pneumoniae* isolates. In Greece, Italy, Portugal, the U.S., and China, *Klebsiella pneumoniae* carbapenemase (KPC) enzymes were the most detected carbapenemases in *K pneumoniae* (2, 3, 7, 10), whereas New Delhi Metallo-β-lactamase (NDM) enzymes were most frequent in Denmark, Montenegro, Serbia, and India (2, 11). According to a 20 Years follow-up of the SENTRY Antimicrobial Surveillance Program (12), the resistance of *K. pneumoniae* to carbapenems increased exponentially over time. In addition, they discovered that the endemicity of ESBLs-encoding genes in *K. pneumoniae* has changed from *bla*_SHV_ *tobla*_CTX−M_ in U.S. hospitals after 2013 (12). Hence, tracking the trends of drug resistance (especially carbapenems) of clinical isolates timely and regionally is essential to prevent the further spread of resistant bacteria and guide the rational use of antibiotics.

In China, bacterial resistance surveillance programs are implemented both regionally and provisionally. There are two national surveillance networks for bacterial resistance: the China Antimicrobial Resistance Surveillance System (CARSS) and the China Antimicrobial Surveillance Network (CHINET). CARSS monitors the different bacterial resistance profiles among disparate provinces and autonomous regions (8). CHINET mainly focuses on the bacterial resistance trends of major referral hospitals based on microdilution methods. The Antimicrobial Resistant Investigation Network of Sichuan Province (ARINSP), established in 2011, is the subordinate network of the China Antimicrobial Resistance Surveillance System (CARSS). It has the responsibility to capture the antibiotic resistance situation in the whole province. The overall resistance rates of different bacteria in different years were reported by CARSS and CHINET (13, 14). However, the resistance profiles of *K. pneumoniae* in Sichuan province were not reported in detail.

Here, we focused on analyzing the distribution of *K. pneumoniae* in different specimen sources and its antibiotic resistance trends from 2017 to 2020, from patients in ARINSP-participating hospitals in China.

## Materials and Methods

### Bacteria Isolates

The bacteria isolates were collected from outpatients and inpatients in ARINSP-participating hospitals from 2017 to 2020, and the annual number of hospitals included in the data analysis was 75, 86, 92, and 92, respectively. According to the monitoring scheme, only one isolate from the same species would be included, and thus the data of non-repetitive isolates were submitted. The isolation criteria of target bacteria from clinical specimens were as follows: (1) all non-contaminated bacteria from sterile site specimens (blood, cerebrospinal fluid, bone marrow, pleural fluid, bladder puncture, urine, ascites, and sterile space puncture fluid tissue); (2) bacteria from qualified specimens of open sites (sputum, pharynx, urine, and feces).

### Identification of Bacteria Species

Species identification of the bacteria was conducted by established methods using the Vitek2 automated system, BD100 system, or matrix-assisted laser desorption ionization-time of flight mass spectrometry.

### Antimicrobial Susceptibility Test

Antimicrobial susceptibility test of the isolates was performed using VITEK2 and BD100 automated systems to determine minimum inhibitory concentrations (MICs). If the drug concentration range of the drug sensitivity test did not cover the cut-off point, a supplementary test was conducted according to the hospitals' clinical needs and the CARSS protocol's requirements (15). The drug sensitivity results confirmed by the additional tests were reported. Antimicrobial susceptibility was confirmed (if necessary, e.g., when imipenem is resistant using VITEK2 system) with the disc diffusion method or E-test. All results were interpreted according to the CLSI document except for tigecycline, which was interpreted according to the FDA criteria. The isolates were tested for ampicillin/sulbactam, cefoperazone/sulbactam, piperacillin/tazobactam, cefazolin, cefuroxime, ceftazidime, ceftriaxone, cefotaxime, cefepime, cefoxitin, ertapenem, imipenem, meropenem, amikacin, gentamycin, ciprofloxacin, trimethoprim-sulfamethoxazole, tigecycline, as recommended by the CARSS.

### Quality Control

According to the CLSI, quality control test was performed routinely once a week. The reference strains were *Klebsiella pneumoniae* (ATCC 700603), *Pseudomonas aeruginosa* (ATCC 27853), and *Escherichia coli* (ATCC 25922).

### Statistical Analysis

The WHONET 5.6 software was used for data analysis. The actual resistance number and rate of each antibiotic were selected for statistical analysis in this monitoring.

## Results

### The Distribution Sites of the Specimen

The specimens' type distribution is shown in Table 1. In total, 833,408 non-repetitive clinical isolates of bacteria were collected during the study period (2017–2020). The bacteria strains isolated from sputum and broncho-alveolar lavage accounted for 48.7, 56.4, 49.2, and 43.7% respectively from year 2017 to 2020. Isolates from urine made up the second population (14.6% in 2017, peaked in 2018 as 17.9%, 15.4% in 2019, and 17.3% in 2020) among all specimen types, followed by blood source (8.7% in 2017, peaked in 2018 as 9.6%, 8.1% in 2019, and 8.6% in 2020) and pus (annually increased from 8.1% in 2017, 9.2% in 2018, 9.4% in 2019, to 10.5% in 2020) (Table 1).

**Table 1:** The distribution of all collected isolates in different specimen sources.

**Specimen type**	**2017 (*****N*** **= 186.585)**		**2018 (*****N*** **= 179.512)**		**2019 (*****N*** **= 236.751)**		**2020 (*****N*** **= 230.560)**	
	***n***	**%**	***n***	**%**	***n***	**%**	***n***	**%**
Sputum and Broncho-alveolar lavage	90.984	48.7	101.163	56.4	116.447	49.2	100.749	43.7
Urine	27.159	14.6	31.930	17.9	36.578	15.4	39.797	17.3
Blood	16.165	8.7	17.170	9.6	19.097	8.1	19.805	8.6
Pus	15.079	8.1	16.462	9.2	22.362	9.4	24.109	10.5
Abscess, abdominal	2.723	1.5	2.741	1.5	3.018	1.3	3.363	1.5
Bile	2.569	1.4	2.605	1.4	2.926	1.2	3.502	1.5
Stool	1.423	0.8	1.387	0.7	1.787	0.8	2.253	0.9
Pleural fluid	7.77	0.4	771	0.4	794	0.3	849	0.3
Cerebrospinal fluid	5.67	0.3	556	0.3	637	0.3	621	0.3
Others	29.139	15.6	4.727	2.6	33.105	14.0	35.512	15.4

*N, the annual total number of all collected isolates; n, the number of each specimen source*.

### The Distribution of *Klebsiella pneumoniae* Among Specimens

The distribution of *K. pneumoniae* among specimens is summarized in Table 2. Although the number of *K. pneumoniae* isolates from sputum and broncho-alveolar lavage increased from 18,809 in 2018, to 19,742 in 2019 and 19,376 in 2020 (the distribution data in 2017 were not available), the proportion was annually decreased from 73.9% in 2018, 66.9% in 2019, to 63.2% in 2020, making up a predominant proportion among all the specimen sources at all times. The proportions of *K. pneumoniae* isolates from urine were found to be 9.9% in 2018, 6.1% in 2019, and 10.5% in 2020. *K. pneumoniae* isolates from blood (7.0% in 2018, 6.6% in 2019, and 7.8% in 2020) and pus (5.2% in 2018, 6.1% in 2019, and 6.7% in 2020) sources increased slightly over time.

**Table 2:** The distribution of *K. pneumoniae* in different specimen sources.

**Specimen type**	**2017 (*****N*** **= 25.119)**		**2018 (*****N*** **= 25.449)**		**2019 (*****N*** **= 29.516)**		**2020 (*****N*** **= 30.678)**	
	***n***	**%**	***n***	**%**	***n***	**%**	***n***	**%**
Sputum and Broncho-alveolar lavage	–	–	18.809	73.9	19.742	66.9	19.376	63.2
Urine	–	–	2.520	9.9	2.931	6.1	3.236	10.5
Blood	–	–	1.791	7.0	1.949	6.6	2.403	7.8
Pus	–	–	1.313	5.2	1.810	6.1	2.062	6.7
Abscess, abdominal	–	–	213	0.8	251	0.9	264	0.9
Bile	–	–	300	1.2	351	1.9	452	1.5
Stool	–	–	56	0.2	83	0.3	126	0.4
Pleural fluid	–	–	60	0.2	63	0.2	77	0.3
Cerebrospinal fluid	–	–	41	0.2	41	0.1	63	0.2
Others	–	–	346	1.4	2.295	7.8	2.619	8.5

*N, the annual total number of all collected K. pneumoniae isolates; n, the isolates number in each specimen source; -, not available*.

### Klebsiella pneumoniae

**Figure 1:**
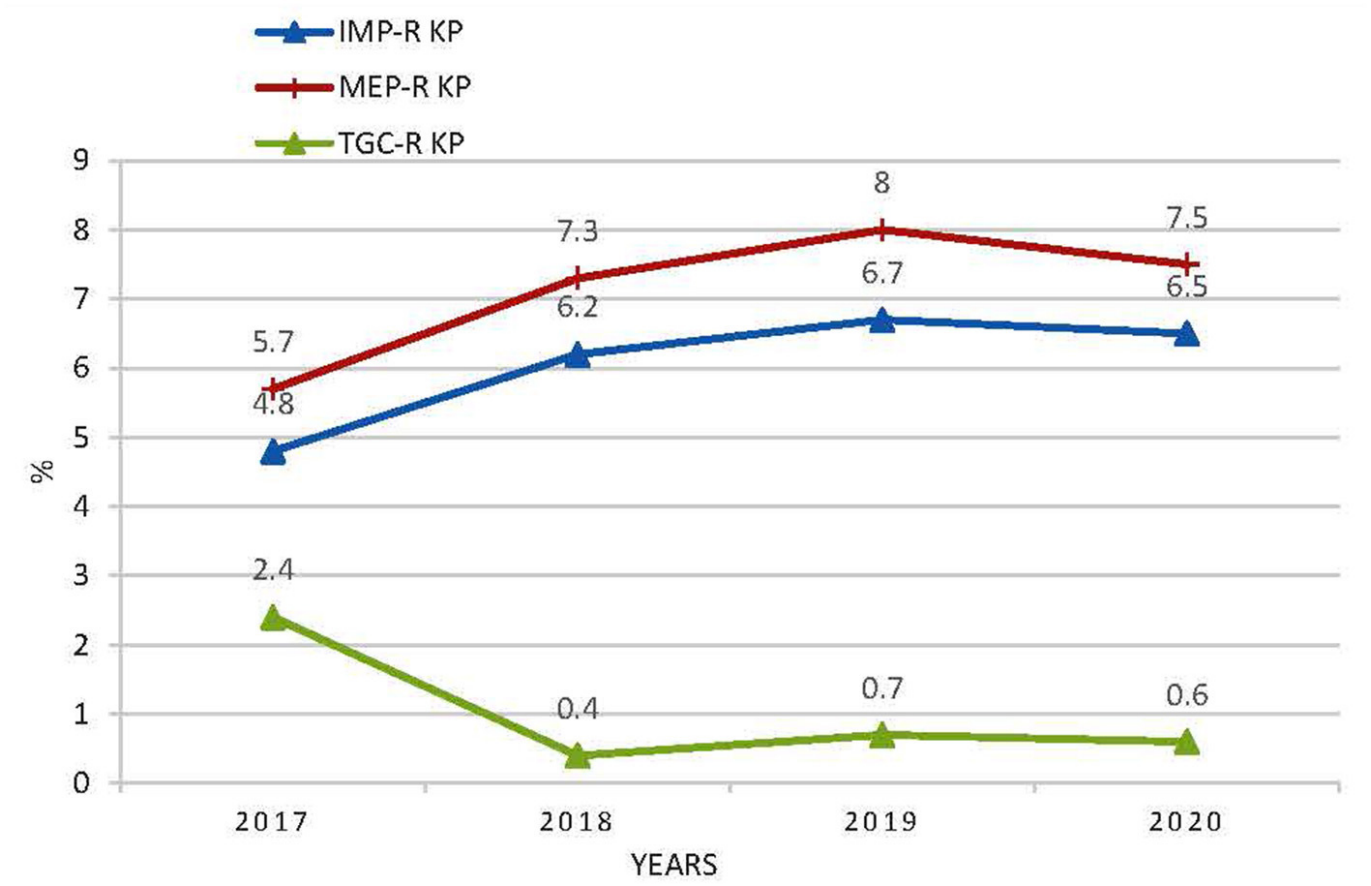
The resistance rates of *K. pneumoniae* to imipenem, meropenem, and tigecycline. IMP KP, imipenem-resistant *K. pneumonia*; MEP-R, meropenem-resistant *K. pneumonia*; TGC-R KP, tigecycline- resistant *K. pneumonia*.

The antimicrobial susceptibility results of *K. pneumoniae* to antibiotics commonly used are shown in Table 3. The tested number of *K. pneumoniae* isolates was 25,115 in 2017, 25,449 in 2018, 29,516 in 2019, and 30,687 in 2020, respectively. The resistance rates of *K. pneumoniae* to ertapenem (2.1% in 2017, 4.3% in 2018, and 3.8% in 2020), imipenem (4.8% in 2017, 6.2% in 2018, peaked in 2019 as 6.7%, and 6.5% in 2020), and meropenem (5.7% in 2017, 7.3% in 2018, peaked in 2019 as 8.0%, and 7.5% in 2020) increased slightly over time (Figure 1). Tigecycline resistance level remained lowest, and the trend declined from 2.4% in 2017 to 0.4% in 2018, and from 0.7% in 2019, to 0.6% in 2020 (Figure 1). A marked increase of resistance was seen for ciprofloxacin from 14.7% in 2017, 15.4% in 2018, 15.5% in 2019, to 26.5% in 2020. The resistance levels of Ampicillin/sulbactam (29.6% in 2017, 29.0% in 2018, 30.0% in 2019, and 29.7% in 2020), ceftazidime (16.8% in 2017, 16.5% in 2018, 16.6% in 2019, and 16.4% in 2020), and cefepime (13.8% in 2017, 13.8% in 2018, 13.3% in 2019, and 13.5% in 2020) were stable during the 4 years. Resistance rate to cefoperazone/sulbactam was found to be 7.0% in 2017, 12.4% in 2018, 12.0% in 2019, and 11.2% in 2020, respectively. The resistance rates of *K. pneumonia* to piperacillin/tazobactam (8.5% in 2017, 8.4% in 2018, 9.0% in 2019, and 9.3% in 2020) and amikacin (2.5% in 2017, 4.6% in 2018, 4.1% in 2019, and 5.0% in 2020) increased slightly over time. The resistance level of *K. pneumoniae* to cefazolin remained highest among all antibiotics tested but decreased from 37.8% in 2017 to 33.8% in 2018 and remained stable in the next 2 years. Resistance rates to ceftriaxone (29.9, 27.2, 26.5, and 25.1% from 2017 to 2020), gentamicin (15.7, 14.9, 14.7, and 14.6% from 2017 to 2020), and trimethoprim-sulfameth (25.4, 24.2, 24.1, and 23.8% from 2017 to 2020) declined slightly over time. Cefuroxime and cefotaxim resistance rates fluctuated around 29.8 and 26.5%, respectively.

**Table 3:** Antibiotic resistance of *Klebsiella pneumonia*.

**Antibiotics**	**2017 (*****N*** **= 25.119)**		**2018 (*****N*** **= 25.449)**		**2019 (*****N*** **= 29.516)**		**2020 (*****N*** **= 30.687)**	
	***n***	***R*%**	***n***	***R*%**	***n***	***R*%**	***n***	***R*%**
Ampicillin/sulbactam	17.398	29.6	22.858	29.0	26.720	30.0	28.241	29.7
Cefoperazone/sulbactam	3.489	7.0	6.123	12.4	9.117	12.0	13.322	11.2
Piperacillin/tazobactam	24.462	8.5	24.796	8.4	28.166	9.0	30.444	9.3
Cefazolin	7.335	37.8	11.069	33.8	15.032	33.2	16.480	33.8
Cefuroxime	8.898	29.7	10.299	31.3	16.431	30.1	18.340	28.1
Ceftazidime	22.899	16.8	23.890	16.5	29.082	16.6	30.167	16.4
Ceftriaxone	18.682	29.9	23.371	27.2	27.858	26.5	30.348	25.1
Cefotaxime	4.612	27.1	4.067	26.7	5.251	25.9	9.404	27.1
Cefepime	20.309	13.8	20.958	13.8	26.162	13.3	28.773	13.5
Cefoxitin	7.648	14.2	8.182	16.1	8.657	16.5	8.447	13.5
Ertapenem	13.678	2.1	16.172	4.3	–	–	23.115	3.8
Imipenem	24.239	4.8	24.984	6.2	28.909	6.7	30.083	6.5
Meropenem	10.365	5.7	11.570	7.3	14.014	8.0	15.162	7.5
Amikacin	24.697	2.5	24.630	4.6	28.988	4.1	30.100	5.0
Gentamicin	24.809	15.7	25.391	14.9	29.219	14.7	28.730	14.6
Ciprofloxacin	23.735	14.7	24.801	15.4	28.782	15.5	24.150	26.5
Trimethoprim-sulfamethoxazole	23.996	25.4	23.969	24.2	28.519	24.1	29.689	23.8
Tigecycline	3.015	2.4	5.652	0.4	8.412	0.7	10.597	0.6

*N, the annual total number of K. pneumoniae; n, the actual number of each antibiotics testing susceptibility, R%, the resistance rates of K. pneumoniae to each antibiotic; -, not available*.

## Discussion

This study aimed to analyze the distribution of *K. pneumoniae* among different specimen sources and its antimicrobial resistance profiles. The annual total number of all collected isolates increased, and specimens from the sputum and broncho-alveolar lavage played the dominant role (48.7% in 2017, 56.4% in 2018, 49.2% in 2019, and 43.7% in 2020) in the study period (Table 1). These results are higher than the domestic level reported by CARSS (14) and the China Antimicrobial Surveillance Network (CHINET) (16). There are still controversies regarding the clinical value of sputum cultures in the management of pneumonia. Saukkoriipi et al. (17) reported that the culture of all sputum samples (either high-quality or low-quality) would add value to the pneumococcal community-acquired pneumonia (CAP)-diagnosis in elderly patients (≥65 years). Another study (18) demonstrated that sputum cultures had no clinical or economic benefits for both CAP and healthcare-associated pneumonia (HCAP) patients. However, cultures can reduce costs and shorten the overall length of hospital stay under some circumstances (e.g., empirical antibiotics therapy). Therefore, clinicians should make decisions based on the traits of patients.

*K. pneumoniae* can cause community-acquired and hospital-acquired infections (HAIs) (19, 20), both of which presents unique challenges for clinicians. In addition, Studies identified *K. pneumoniae* pathogens as a leading cause of HCAP (21, 22). In this study, the most common source of *K. pneumoniae* isolates was sputum and broncho-alveolar lavage (73.9% in 2018, 66.9% in 2019, and 63.2% in 2020), followed by urine (9.9% in 2018, 6.1% in 2019, and 10.5% in 2020), blood (7.0% in 2018, 6.6% in 2019, and 7.8% in 2020), and pus (5.2% in 2018, 6.1% in 2019, and 6.7% in 2020) (Table 2). The distribution and drug susceptibility profiles of *K. pneumoniae* in community-acquired infections and HAIs could be further analyzed if the information of outpatients and inpatients was available.

*K. pneumoniae*, which belongs to the *Enterobacteriaceae*, is one of the most threatening pathogens and a significant source of antibiotic resistance (23). In the last decade, CRE has spread rapidly and caused great public health concerns (24), of which CRKP was one of the most important pathogens. A study (13) reported that the rate of CRKP was increased from 2.9% in 2005 to 10.0% in 2012 and 25.3% in 2019, an ~8-fold increase. Moreover, the rate of CRKP rose from 0.7 to 14.2% in Europe and from 0.5% to 6.1% in APAC during 1997–2016 (25), yet the rate of CRKP (meropenem, 5.7% in 2017, 7.3% in 2018, peaked in 2019 at 8.0%, 7.5% in 2020, Table 3) in Sichuan province was much lower than the domestic level (13). Europe was reported (25) with both increased CRKP's number and enhanced resistance rate. This scenario presents significant challenges for clinicians. Although some countermeasures such as guidelines (26) and surveillance networks were applied to curb these pathogens, the result remains dissatisfied. The reasons for the failure in curbing CRKP are not well-understood (21). However, several critical factors, such as the overcrowding and shortage of staff, the excessive use of carbapenems, and the absence of a network to share patient information, may contribute to their spread. Further measures should be taken to curb the spread. Additionally, with the rapid increase in CRKP prevalence, antibiotic treatment therapy for CRKP is extremely limited in clinical practice. Ceftazidime-avibactam, meropenem-vaborbactam, imipenem-cilastatin-relebactam, cefiderocol, or tigecycline were considered the last line agents for treating infections caused by CRE (27). Only ceftazidime-avibactam and tigecycline are marketed in China. Ceftazidime-avibactam, first approved by the US Food and Drug Administration (FDA) in 2015 (28) and marketed in China in 2019 (29), is a promising drug for treating infections caused by carbapenem-resistant gram-negative bacilli (28, 30, 31). However, it developed resistance rapidly (30), further diminishing the limited options for antibiotic treatments. Therefore, the microbiological laboratory staff should contact the clinical to add potentially practical antibiotic tests (e.g., ceftazidime-avibactam, tigecycline) once CRKP is detected.

The resistance rate of *K. pneumoniae* to tigecycline remained lowest among all tested antibiotics, which declined from 2.4% in 2017 to 0.4% in 2018 and from 0.7% in 2019 to 0.6% in 2020, suggesting that tigecycline has retained high activity over *K. pneumoniae*. These results were lower than the tigecycline resistance level in Europe (5% according to its EUCAST recommended breakpoint) (2). However, the microbiological laboratory technicians should notice that when tigecycline susceptibility was moderately sensitive or resistant (measured by paper dispersion or automated systems method), an additional test using the micro broth dilution method should be conducted to confirm the susceptibility. Many factors can affect the *in vitro* activity of tigecycline, such as the media type, medium detection method, and breakpoint selection (32). Currently, the underlying resistance mechanisms of *K. pneumoniae* to tigecycline have not been fully understood (33, 34). However, it is mainly related to the upregulation of resistance-nodulation-division (RND) efflux pump AcrAB and OqxAB, which was regulated by the mutations of transcriptional genes *ramR* and *acrR* and the upregulation of *ramA* (35, 36)*, acrB, rarA*, and *oqxB* (33).

A marked increase of resistance to ciprofloxacin was noted from 14.7% in 2017, 15.4% in 2018, 15.5% in 2019, to 26.5% in 2020, similar to the trends (from 7.3% in 1997 to 27.9% in 2016) reported by the SENTRY Antimicrobial Surveillance Program (12). The *bla*_CTX−M_ gene was demonstrated to be responsible for the increased resistance to ciprofloxacin in US hospitals. Besides, *bla*_CTX−M_ ESBL is the most common genotype in China (37). Urgent measures should be taken to reserve the drug susceptibility.

Our study has several limitations. Firstly, due to patients' information not being available, we did not analyze the antimicrobial resistance rates among outpatients and inpatients. Secondly, not all hospitals conform to the standards (e.g., personnel, equipment, facilities, methodology) to participate in the ARINSP Program to ensure monitoring accuracy. Therefore, we are not able to capture all in this study. Thirdly, the testing methods used in some hospitals are not identical. Uniformity of the methodology applied in some hospitals is not there that may affect the result.

In conclusion, the increasing trend of *K. pneumoniae*'s antimicrobial resistance to carbapenems exists, while tigecycline has retained activity to against *K. pneumoniae*. Since the resistance mechanisms of *K. pneumoniae* could be different in various populations from different regions (38), future surveillance is essential. It can help for implementing intervention programs/plans to reduce the occurrence of antimicrobial resistance and to provide with a rational use of antimicrobials.

## Data Availability Statement

The original contributions presented in the study are included in the article/supplementary files, further inquiries can be directed to the corresponding author/s.

## Ethics Statement

The study was conducted on retrospective data. Ethical approval was obtained from the Institutional Review Board of Sichuan Provincial People's Hospital, and University of Electronic Science and Technology of China (Number: 2021-511).

## Author Contributions

HY designed the study. JZ and DL contributed to manuscript writing, revised, and supervised the project. SL and XH checked the data. All authors contributed to the article and approved the submitted version.

## Funding

This work was supported by the National Natural Science Foundation of China (81702064).

## Conflict of Interest

The authors declare that the research was conducted in the absence of any commercial or financial relationships that could be construed as a potential conflict of interest.

## Publisher's Note

All claims expressed in this article are solely those of the authors and do not necessarily represent those of their affiliated organizations, or those of the publisher, the editors and the reviewers. Any product that may be evaluated in this article, or claim that may be made by its manufacturer, is not guaranteed or endorsed by the publisher.
